# Crushing Response and Optimization of a Modified 3D Re-Entrant Honeycomb

**DOI:** 10.3390/ma17092083

**Published:** 2024-04-28

**Authors:** Jun Zhang, Bo-Qiang Shi, Bo Wang, Guo-Qing Yu

**Affiliations:** 1 School of Mechanical Engineering, University of Science and Technology Beijing, Beijing 100083, China; b20180266@xs.ustb.edu.cn; 2 Xinxing Cathay International Intelligent Equipment Technology Research Institute Co., Ltd., Beijing 100071, China; wangbo_201@126.com; 3 State Key Laboratory of Intelligent Manufacturing of Advanced Construction Machinery, Xuzhou 221004, China; yukuoching_ustb@163.com

**Keywords:** re-entrant honeycombs, crushing response, energy absorption, optimization

## Abstract

A modified 3D re-entrant honeycomb is designed and fabricated utilizing Laser Cladding Deposition (LCD) technology, the mechanical properties of which are systematically investigated by experimental and finite element (FE) methods. Firstly, the influences of honeycomb angle on localized deformation and the response of force are studied by an experiment. Experimental results reveal that the honeycomb angles have a significant effect on deformation and force. Secondly, a series of numerical studies are conducted to analyze stress characteristics and energy absorption under different angles (α) and velocities (v). It is evident that two variables play an important role in stress and energy. Thirdly, response surface methodology (RSM) and the Non-Dominated Sorting Genetic Algorithm II (NSGA-II) are implemented with high precision to solve multi-objective optimization. Finally, the final compromise solution is determined based on the fitness function, with an angle of 49.23° and an impact velocity of 16.40 m/s. Through simulation verification, the errors of energy absorption (EA) and peak crush stress (PCS) are 9.26% and 0.4%, respectively. The findings of this study offer valuable design guidance for selecting the optimal design parameters under the same mass conditions to effectively enhance the performance of the honeycomb.

## 1. Introduction

Various types of systematically designed artificial materials, known as mechanical meta-materials, possess the capability to exhibit mechanical characteristics and responses that surpass those of their base materials. These materials have attracted significant research interest owing to their exceptional mechanical properties in comparison to traditional materials, including superior shear resistance [1], indentation resistance [2,3], fracture resistance [4], blast resistance [5,6], synclastic behavior [7], and energy absorption [8,9]. The remarkable mechanical features possess diverse potential applications for auxetic in various engineering fields, such as biomedical implants [10], flexible electronics [11], morphing wings [12], nails [13], and automobile energy absorption devices [14]. Currently, there are several types of auxetic mechanical meta-materials, including concave structures [15] and chiral structures [16]. Concave structures are primarily divided into three models: the re-entrant model [17,18], double arrow model [19], and star model [20]. Researchers are paying more attention to the re-entrant model in auxetic structures that use concave designs because it is easier to fabricate and has better auxeticity than other types of auxetic units. Under a variety of loading scenarios, auxetic honeycombs featuring a re-entrant design have outperformed traditional convex hexagonal honeycombs in terms of mechanical performance [21].

Re-entrant honeycombs are a popular form of auxetic meta-materials extensively employed as core components in sandwich panels to withstand dynamic loads [22]. Leveraging the re-entrant mechanism, various innovative structures have emerged. For example, Wang et al. [23] used theoretical and computational techniques to study the in-plane impact reactions of a re-entrant star-shaped honeycomb. The results of the FE study show that this honeycomb has better impact resistance than its classical re-entrant cousin with the same thickness of the cell wall. Notably, the transverse contraction primarily occurs at the initial plateau region, a distinct behavior from the classical re-entrant honeycomb. By substituting double circular arc cell walls for the inclined cell walls of the re-entrant unit, Qi et al. [24] created a unique re-entrant circular honeycomb and investigated its in-plane crushing response using experimental, computational, and theoretical methods. The suggested honeycomb’s specific energy absorption was found to be much higher than that of the conventional re-entrant honeycomb, according to the results. More plastic hinges were formed as a result of the double arc walls, which improved energy dissipation during the crushing process. The modification led to a significant rise in energy absorption when compared to the standard honeycomb structure. Zhu et al. [25] presented an innovative re-entrant honeycomb with zigzag inclined ligaments that could be produced economically using a conventional fabrication technique. The suggested honeycomb provides increased rigidity while retaining its natural auxetic characteristics, according to the results. Furthermore, Ma et al. [14] proposed a novel dual-functional meta-material that integrates a re-entrant honeycomb structure featuring square cells and conducted a theoretical and numerical study into energy absorption capacity of this meta-material. The resulting hierarchical re-entrant honeycomb meta-material exhibits significantly enhanced energy absorption and outstanding vibration insolation capabilities.

Multi-objective optimization is typically used to balance two conflicting optimization objectives, but balancing these objectives proves challenging due to their inherent contradiction. Nevertheless, this optimization method has demonstrated effectiveness in similar problems. Tan et al. [26] utilized the NSGA-II and Archive-based Micro Genetic Algorithm (AMGA) to effectively optimize the crashworthiness and enhanced the crushing performance of a novel re-entrant hierarchical crash box. The optimal Latin hypercube design method and the accurate adaptive Kriging model were used to improve the energy-absorbing capabilities of honeycomb structures across a range of amplitudes and periods [27]. The algorithm was then used to conduct an optimization for the sinusoidal honeycomb construction. Finally, the optimized results demonstrated a 30.53% reduction in peak crush force for the sinusoidal honeycomb structure, coupled with a 38.55% increase in specific energy absorption. Jiang et al. [28] performed a comparison between the conventional re-entrant core and the re-entrant circle core, both possessing the same mass. The results showed that the re-entrant circle core can improve the system’s energy absorption by 5.7% and reduce the maximum displacement of the rear panel by 2.2%. Following multi-objective optimization, the maximum displacement of the graded core sandwich panel was further reduced by 33.0%. The NSGA-II technique was applied for a multiple objective investigation on graded foam-cored and uniform sandwich panels by Wang et al. [29]. The outcomes demonstrated that the best solutions obtained from the Pareto set had better blast resistance and less mass than the baseline design. An optimization investigation on the re-entrant honeycomb cored sandwich panel was carried out by Qi et al. [30]. The outcomes showed that, in contrast to conventional optimization, the optimization led to a 4.9% improvement in specific energy absorption and a 1.8% improvement in maximum displacement. 

The collective findings from the aforementioned studies show that numerous researchers have illustrated the diverse responses of honeycombs under different loads, which vary with the honeycomb’s structural configuration. To fully investigate the outstanding mechanical properties of re-entrant honeycomb structures, this study proposes a novel 3D re-entrant honeycomb, which establishes a parametric model with different angles. To investigate the effectiveness of the suggested meta-materials, the FE approach and an experimental in-plane compression test are utilized. Along with exploring the behavior of the provided meta-materials under various loading circumstances, parametric studies are conducted to gain a deeper understanding of the impact of geometric parameters on the behavior of the meta-materials. Subsequently, utilizing a full factorial design (FFD) method combined with RSM, a multiple objective optimization of the modified 3D re-entrant honeycomb is conducted by NSGA-II. Ultimately, the Pareto sets of the model are derived, and optimal solutions are derived within specified constraints of the honeycomb.

## 2. Specimen Preparation and Testing

### 2.1. Geometry of Modified 3D Re-Entrant Honeycombs

**Figure 1:**
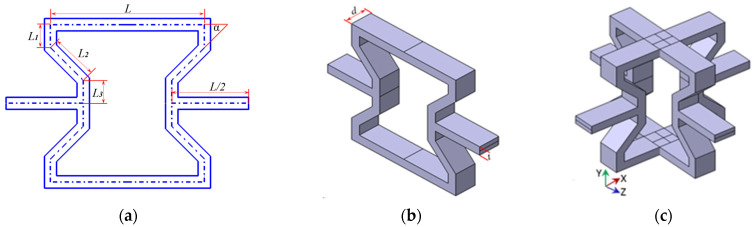
The evolution of the representative unit: (**a**) the dimension of the 2D cross section, (**b**) the schematic diagrams of the 2D representative unit, (**c**) the schematic diagrams of the 3D representative unit.

The representative unit of modified 3D re-entrant honeycomb is formed by a 2D honeycomb vertically aligned in space. The evolution of the representative unit is seen in Figure 1. The cross-section dimensions of the 2D honeycomb are shown in Figure 1a. It is shown that the 2D re-entrant honeycomb’s oblique truss becomes three segments, with lengths of *L*_1_, *L*_2,_ and *L*_3_, respectively. In addition, the bottom and top trusses are both L in length. Half of the top truss is the connect truss with a length of L/2, and the angle between the top truss and the oblique truss is α. Figure 1b manifests the 2D honeycomb, which is vertically aligned to assemble the 3D honeycomb, shown in Figure 1c, where the out-of-plane depth of the 2D honeycomb is d and the thickness of the 2D honeycomb is t.

The relative density of the modified 3D re-entrant honeycomb according to the porous materials theory is determined by comparing the actual volume of representative units to their three-dimensional spatial volume. So, the relative density can be calculated using the following formula:

(1) $$\Delta \rho =\frac{\rho^{*}}{\rho }=\left(\frac{t}{L}\right)\frac{d(2L+4L_{1}+4L_{2}+4L_{3}-d/2)}{{\left[2\left(L-L_{2}\cos\alpha \right)\right]}^{2}\left(L_{1}/L+L_{3}/L+\sin\alpha \right)}$$

where ρ* is the actual density of representative units, and *ρ* is the density of the 3D spatial volume, which refers to the density of the base material in this article.

### 2.2. Experimental Test of Modified 3D Re-Entrant Honeycombs

**Figure 2:**
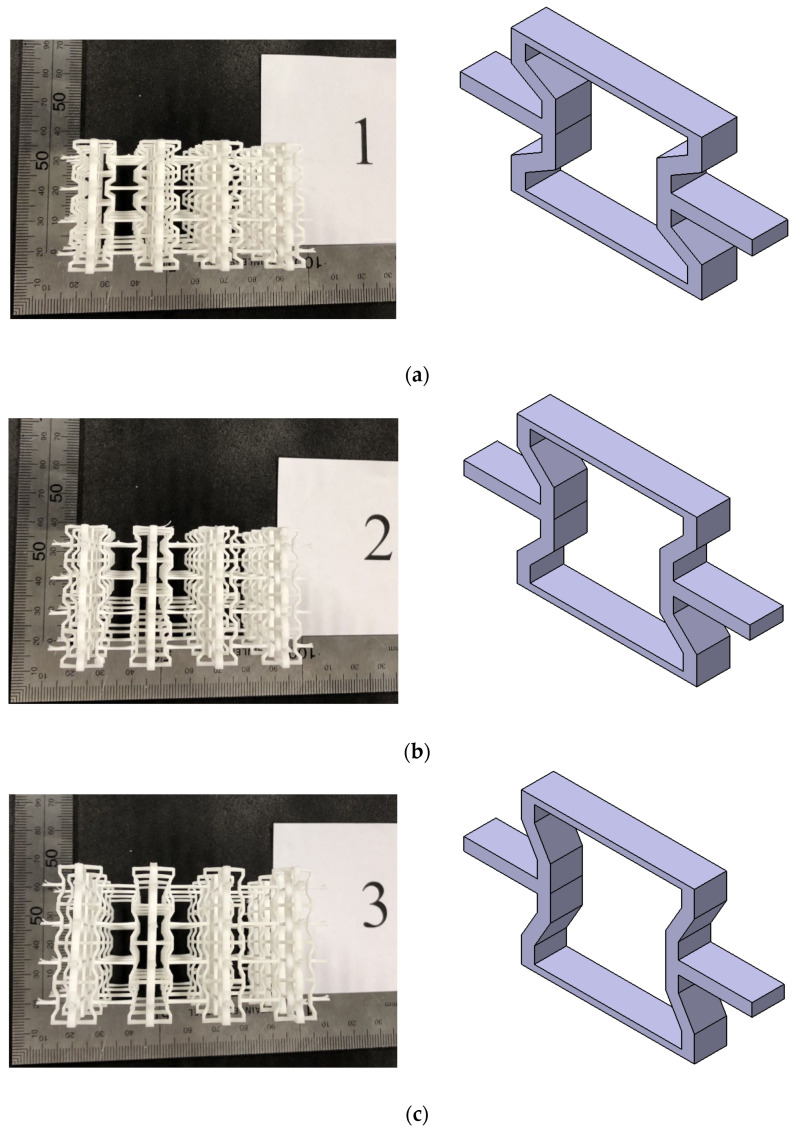

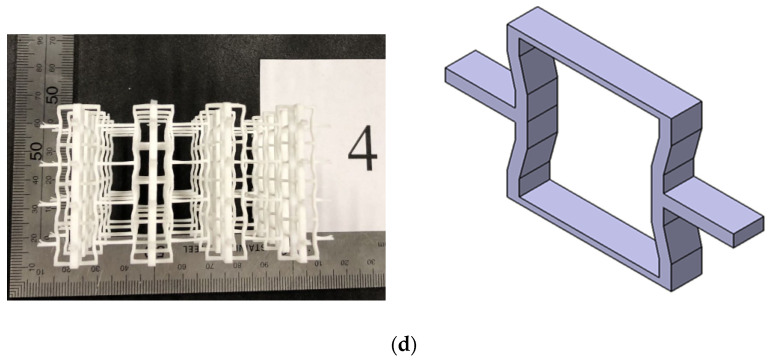
The sample of 3D re-entrant honeycomb: (**a**) sample with a 30° angle, (**b**) sample with a 45° angle, (**c**) sample with a 60° angle, (**d**) sample with a 75° angle.

The modified 3D re-entrant honeycomb structure is relatively intricate, making it challenging to produce using conventional manufacturing methods. Consequently, four specimens depicted in Figure 2, each featuring distinct angles (α), were manufactured utilizing a 3D printer (LD-006, CREALITY, Longhua District, Shenzhen, China) equipped with LCD technology. The overall dimensions of the printer are 325 mm × 290 mm × 500 mm, while the maximum print size is 192 mm × 120 mm × 250 mm, the layer thickness is 0.05 mm, the machine printing orientation is from top to bottom, and the infill percentage of each truss in the honeycomb is 100%. In addition, the printer has a maximum speed of 60 mm per hour and a printing precision of 0.01 mm. The specimen with width L_1_ = L_3_ = 1.5 mm, L_2_ = 2 mm, d = 2 mm, t = 0.8 mm, and L = 10 mm and the angles of α = 30°, α = 45°, α = 60°, and α = 75°, respectively, is shown in Figure 2. The above parameters and the total dimensions of the honeycomb contour profile (L_x_, L_z_, and L_y_) are shown in Table 1. The printing material of the four samples is ABS resin, which is supported by Zhongshan Huayu Yuanxing Electronic Technology Co., Ltd. (Zhongshan, China). The property of the material is given in Section 4.1.

**Figure 3:**
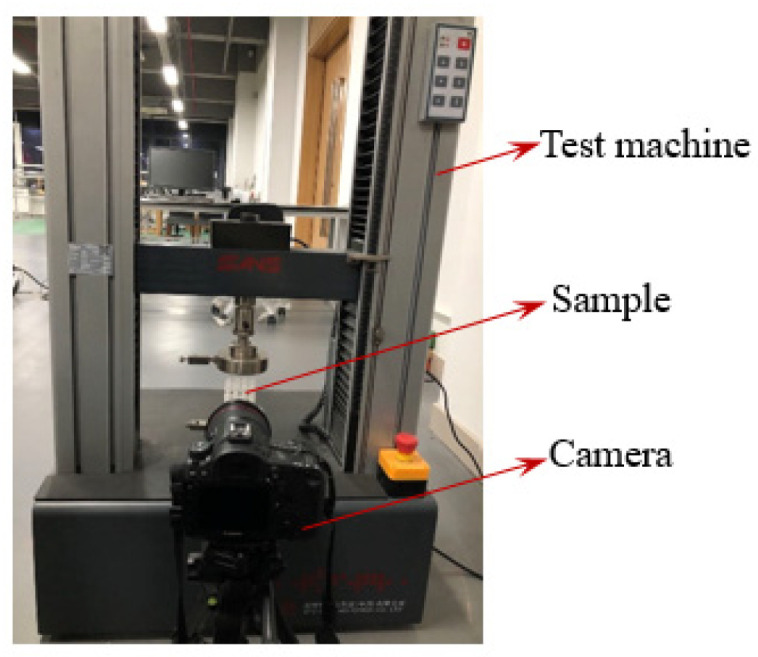
The system of the quasi-static test.

A quasi-static compression test was conducted on the modified 3D re-entrant honeycomb using a universal testing apparatus (CMT4304) provided by MTS Systems (Longhua New District, Shenzhen, China) Co. Ltd., the maximum force of the equipment is 30 kN, the maximum power is 850 watts, and the accuracy is controlled within 0.5%. As shown in Figure 3, the sample was placed unrestricted on a flat steel plate that was fastened in the bottom and subsequently compressed at a loading speed of 5 mm per minute by a top steel plate. The crushing force was recorded by the sensor of the device, and the sample’s deformation process was simultaneously captured by a high-definition digital camera.

## 3. Result of Quasi-Static Experiment

The purpose of this section is to investigate the quasi-static compression behavior of four different angles of 3D modified re-entrant honeycomb, mainly studying the deformation mode and crushing force of the honeycomb during the compression process.

### 3.1. Deformation of Four Modified 3D Re-Entrant Honeycombs with Different Angles

**Figure 4:**
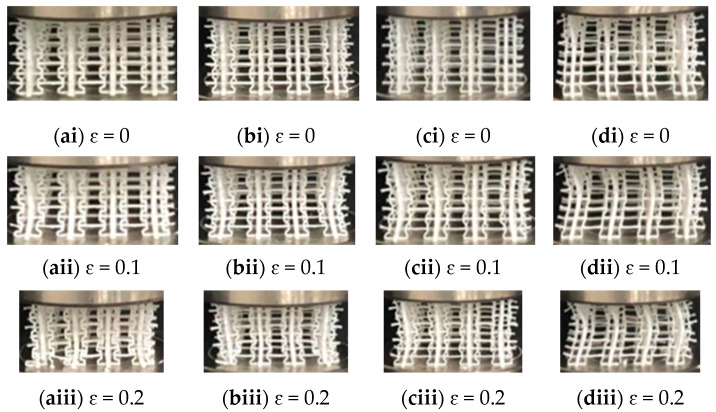
Deformation of four modified 3D re-entrant honeycombs with different angles: (**ai**–**aiii**) the deformation of the 30° sample; (**bi**–**biii**) the deformation of the 45° sample; (**ci**–**ciii**) the deformation of the 60° sample; (**di**–**diii**) the deformation of the 75° sample.

Distinct deformation patterns were observed in 3D modified re-entrant honeycombs from four different angles, revealing various phases during the compression experiment. As shown in Table 1, the angle of the honeycomb (α) increases from 30° to 75° in correspondence with the increases in height of the honeycomb on the y-axis (L_y_), which range from 32.0mm to 39.456 mm, and both the increases in the width of the honeycomb on the x-axis (L_x_) and the width of the honeycomb on the z-axis (L_z_), which range from 66.144 mm to 75.86 mm. Regardless of the angle of the honeycomb, under a continuous compression of 5 mm per minute in the y-axis direction, the honeycomb structure shrinks from top to bottom and also experiences corresponding shrinkage in the horizontal direction (x-axis and z-axis), as sown in Figure 4. When the strain reaches 0.1, each angle of the honeycomb exhibits a negative Poisson’s ratio (NPR) phenomenon in the shape of ‘) (’. According to the deformation of each honeycomb with a strain of 0.2, it can be observed in Figure 4 that when the honeycomb angle is 30°, there is obvious fracture or crushing in the lower part of the honeycomb, but when the honeycomb angle is increased to 75°, there is fracture or crushing in the upper part of the honeycomb. This indicates that with the increase in the honeycomb angle, due to the increase in porous voids, under the same external compression conditions, the force is less likely to be transmitted to the lower part of the honeycomb. Additionally, with the increase in the honeycomb angle, the truss inside the honeycomb structure becomes more unstable in the quasi-static test and the negative Poisson’s ratio phenomenon becomes more pronounced.

### 3.2. Crushing Force of Four Different Angle Modified 3D Re-Entrant Honeycombs

**Figure 5:**
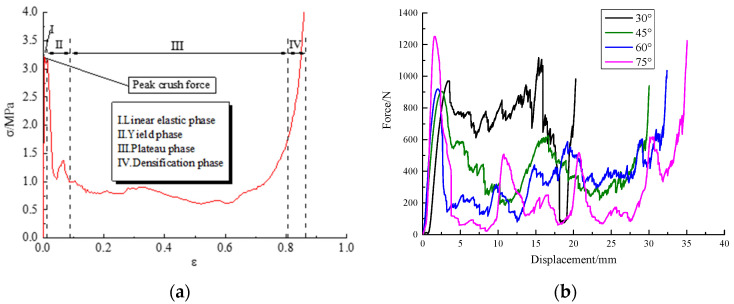
Division of stress regions and force–displacement curves of four modified 3D re-entrant honeycombs: (**a**) phase division of the stress–strain curve; (**b**) the force–displacement curve of four different honeycombs.

In general, the compression process can be divided into four phases, as shown in Figure 5a. The maximum force observed during the linear elastic phase is termed peak crush force. Due to the different angles of the four modified re-entrant honeycombs, even if the length of each truss in the honeycomb is the same—that is the same mass—the height of the honeycomb along the y-axis direction is not the same. Consequently, each phase of honeycomb exhibits distinct compressive displacements. In the linear elastic phase, four different honeycombs have different compression distances, as shown in Figure 5b. When the angle is 30°, the displacement is 3 mm in this state. When the degree of angle is 75, the displacement in this period is only 2 mm. As the honeycomb angle increases, the compression displacement continuously decreases, meaning that the elastic phase is completed faster. The plateau phase and the density phase also have the same characteristics. With the honeycomb angle increase from 30° to 75°, the densification phases begin from 20 mm, 28 mm, 32 mm, and 35 mm, respectively. This is because although the L_2_ truss has the same length, its height in the y-axis direction varies with the angle α, as shown in Table 1. With the angle increase, the plateau force of the 3D modified re-entrant honeycombs decreases from about 800 N to 300 N. In addition, the force becomes more fluctuating. It is concluded that with the angle increase, modified 3D re-entrant honeycombs have a longer platform phase with lower force. Further, the changes in the modified 3D re-entrant honeycombs’ absorption energy through simulation analysis need to be further studied.

## 4. Numerical Analysis

### 4.1. Parameter Settings for the Finite Element Model

**Figure 6:**
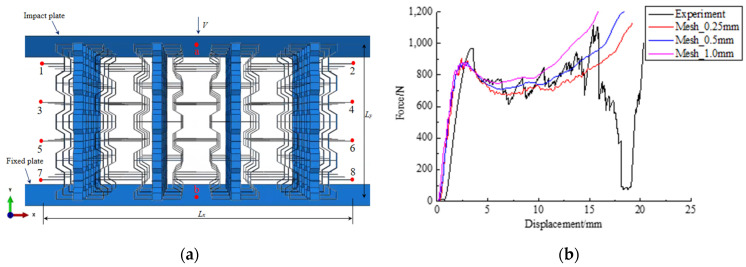
FE model establishment and mesh size selection: (**a**) the establishment of the FE model; (**b**) comparison of different mesh sizes and experiment.

The commercial software Abaqus/Explicit 6.14 finite element method was used to carry out a nonlinear dynamic explicit analysis in this work. Figure 6a presents the finite element (FE) model, illustrating the impact on the 3D modified re-entrant honeycomb structure. The honeycomb specimen features a width denoted as L_x_, a height as L_y_, and an out-of-plane dimension L_z_ equal to its width L_x_, which is placed in the middle of two rigid plates for analysis. The lower and upper plates are set as analytic rigid. The lower plate is fastened while the sample was impacted by the upper plate at an invariable velocity v along the y-axis direction. The x-axis and z-axis are maintained in a free state at the same time. To effectively capture the force curve of the sample, the honeycomb structure is configured with four cells in both the x- and y-axes, and four cells in the z-axis for out-of-plane deformation, respectively. The honeycomb material is modeled as elastic and fully plastic, the mass density is 1.04 g/cm^3^, the Young’s modulus is 2500 MPa, the Poisson’s ratio is 0.3, and the yield stress is 50 MPa. To ensure computational accuracy and convergence, the honeycomb structure is simulated using a 4-node quadrilateral finite film strain linear reduction integral shell element (S4R). Furthermore, five integration points are established along the truss thickness direction. Through iterative convergence analysis, a generic contact algorithm is employed between the honeycomb structure and the two plates. All surfaces of the honeycomb susceptible to penetration during compression are designated as self-contact surfaces. The contact friction coefficient between the plates and the specimen is set to 0.3 [31].

### 4.2. Verification of Finite Element Modelling

The experimental quasi-static compression test is carried out using the universal testing machine CMT 4304 as indicated in Figure 6b in order to verify the accuracy of the finite element model. The ABS plastic 4 × 4 × 4 cells in the x-, y-, and z-axes fabricated by LCD3D printing are used in the experimental specimen, the corresponding values of L_1_ = L_3_ = 1.5 mm, L_2_ = 2 mm, L = 10 mm, t = 0.8 mm, and d = 2 mm were adopted for the cells, respectively. The sample is positioned on the lower plate, while the upper plate is crushing at a constant compression velocity of 5 mm per minute. In order to compare the simulation with the experiment, it is also run under identical conditions. The results of the FE simulations and experiment are presented in Figure 6b.

It is observed from the force–displacement curves, with the finite mesh size increase from 0.25 mm to 1.0 mm, the force decreases from the displacement of 2.0 mm. The production procedure is thought to have introduced a tiny flaw in the experimental sample, which could have an impact on the peak load and drop pattern, as shown in Figure 5b. When the displacement is within 2 mm, the stress situation of the three models with different mesh sizes remains basically consistent. Upon surpassing 2 mm, three mechanical models with different mesh sizes exhibit changes in force response. When the displacement exceeds 15 mm, the sample fractures. Among them, at the same displacement, the force is minimal when the mesh size is 0.25 mm, followed by the force observed with a 0.5 mm mesh size, while the maximum force is encountered with a 1.0 mm mesh size. The result with a mesh size of 0.5 mm is closer to the experiment, albeit with a slight deviation from the experimental data. Consequently, it is decided that the mesh size is 0.5 mm. Therefore, the ability of FE models to forecast the honeycomb’s crushing performance is trustworthy.

### 4.3. Parametric Studies

The aim is to research several factors’ capacity to absorb energy throughout the impact resistance process of this kind of honeycomb construction in a practical application. The honeycomb’s material is set to aluminum, and its material properties are taken to be rate-independent elastic perfect plastic with a density of 2700 kg/m^3^, a Young’s modulus of 68 GPa, a yield strength of 255 MPa, a Poisson’s ratio of 0.3, and a friction set to 0. The remaining settings match the above verified simulation circumstances.

#### 4.3.1. Poisson’s Ratio of Modified 3D Re-Entrant Honeycomb with Different Angles

**Figure 7:**
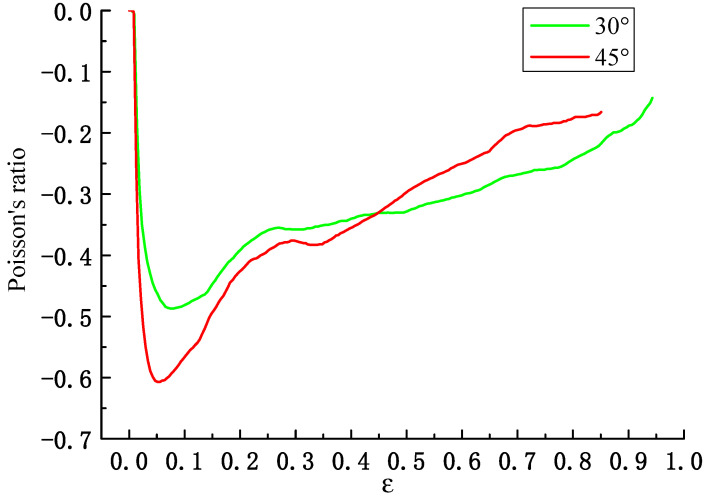
Poisson’s ratio of modified 3D re-entrant honeycomb at 30° and 45°.

Poisson’s ratio is an important indicator for characterizing the deformation process of the honeycomb. This article calculates the Poisson’s ratio through Formula (2) by reasonably selecting 10 landmark points, namely points 1–8 and a, b, as shown in Figure 6a. It can be seen from Figure 7 that the Poisson’s ratio varies with strain. Specifically, the value of Poisson’s ratio decreases from zero and then increases, and the minimum Poisson’s ratio of two honeycombs at 30° and 45° are −0.48 and −0.62, respectively. When the angle of honeycomb is larger than 60°, the honeycomb will collapse and cause sliding during the compression process, so the passion’s ratios have significant change and loss of accuracy. It can be concluded that the results agree with the experiment in Section 2; as the angle of the honeycomb increases, it is more likely to cause a negative Poisson’s ratio phenomenon.

(2) $$\nu =-\frac{\varepsilon_{x}}{\varepsilon_{y}}=-\frac{\delta_{x}\times L_{y}}{\delta_{y}\times L_{x}}=-\frac{\left(\delta_{1–2}+\delta_{3–4}+\delta_{5–6}+\delta_{7–8}\right)\times L_{y}}{4\times \delta_{a–b}\times L_{x}}$$

#### 4.3.2. Stress–Strain Result of Modified 3D Re-Entrant Honeycomb with Different Angles

**Figure 8:**
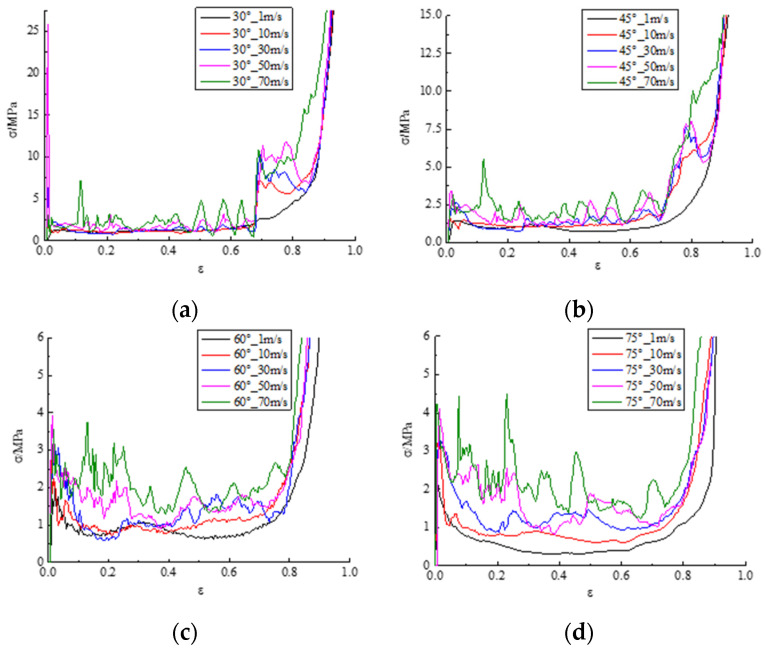
Comparison of stress–strain curves of honeycomb with four angles at different velocities: (**a**) the stress–strain curve of the 30° sample; (**b**) the stress–strain curve of the 45° sample; (**c**) the stress–strain curve of the 60° sample; (**d**) the stress–strain curve of the 75° sample.

The formulas to obtain the results of stress and strain caused by impact in the y-axis for the modified 3D re-entrant honeycomb are follows:

(3) $$\sigma =\frac{F}{L_{x}\times L_{z}}$$

(4) $$\varepsilon =\frac{\delta_{a–b}}{L_{y}}$$

where *F* represents the reacting force of the upper plate, *L_x_*, *L_y_*, and *L_z_* denote the overall dimensions of the modified 3D re-entrant honeycomb in the direction of x-, y-, and z-axes, respectively. *δ* is the displacement at a certain moment along the y-axis. Based on Formulas (3) and (4), Figure 8 displays the stress–strain curves of the honeycomb with different angles (30°, 45°, 60°, and 75°) through the movement of the upper plate from top to bottom at five impact velocities (1 m/s, 10 m/s, 30 m/s, 50 m/s, and 70 m/s). As depicted in this Figure, the modified 3D re-entrant honeycomb exhibits a dynamic response pattern similar to that of a traditional honeycomb during the impact condition, characterized by the linear elastic phase, plateau phase, and densification phase. The stress–strain curves in Figure 8 indicate that, as the honeycomb angle remains constant, the amplitude of stress fluctuations significantly increases with the increasing impact velocity. Additionally, the magnitude of stress gradually increases during the plateau phase. For honeycomb angles of 30° in Figure 8a and 45° in Figure 8b, a secondary stress plateau is observed within the strain range of 0.7–0.9. The values of the second plateau are at least three times that of the first plateau. This phenomenon is associated with the significant NPR effect occurring at a certain height when the honeycomb compresses from the y-axis direction under these two angular conditions. As the honeycomb angle increases, the stress gradually decreases at the same velocity. Furthermore, it is evident that when the honeycomb angle is 45° in Figure 8c, the maximum impact stress is relatively low under different velocity conditions, implying a lower maximum impact force. These findings provide a data foundation and direction for the subsequent optimization of this paper.

#### 4.3.3. Energy Absorption of the Modified 3D Re-Entrant Honeycomb with Different Angles 

**Figure 9:**
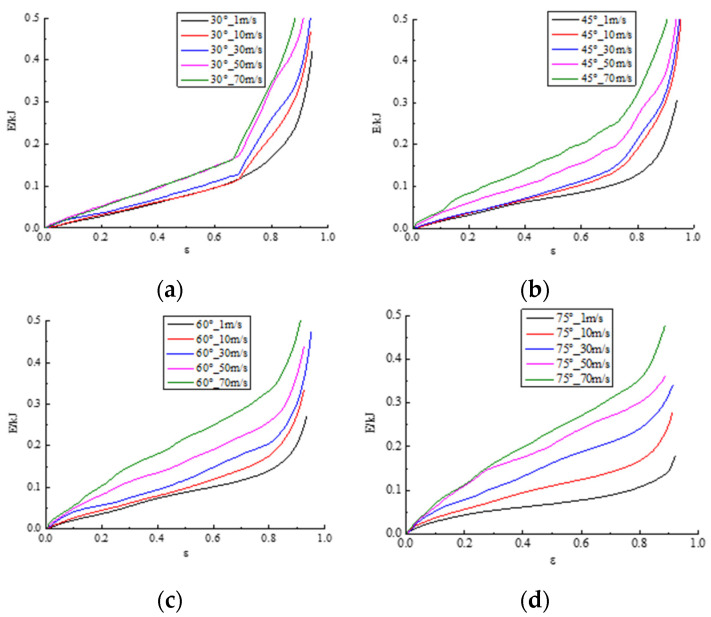
Comparison of energy–strain curves of honeycomb with four angles at different velocities: (**a**) the energy–strain curve of the 30° sample; (**b**) the energy–strain curve of the 45° sample; (**c**) the energy–strain curve of the 60° sample; (**d**) the energy–strain curve of the 75° sample.

It is evident that in Figure 9, with the increase in strain (displacement), each energy–strain curve is a concave curve as the honeycomb structure is compressed. The absorbed energy remains relatively steady during the impact process of the linear elastic phase, yield phase, and platform phase. As a result, the curves almost form oblique lines passing through the zero point. However, when the densification phase approaches, there is a significant increasing trend in the energy–strain curve, resulting in a bending inflection point upon entering the densification stage. As the honeycomb angle remains constant, the energy–strain curves increase with the rise in impact velocity. The arrangement of the curves is sorted from top to bottom based on the magnitude of the velocity. This phenomenon is particularly evident when the cell angle is 60°, as shown in Figure 9c, and 75°, as shown in Figure 9d. This is due to the higher relative porosity of the honeycomb structure, as the angle in the density of the structure decreases proportionally. The greater porosity enables the honeycomb structure to absorb more energy.

## 5. Optimization of Modified 3D Re-Entrant Honeycomb

Peak crush stress (PCS) and energy absorption (EA), two properties of the modified 3D re-entrant honeycombs, directly fluctuate with changes in angle and impact velocity, according to Section 4.3 of the dynamic response and energy absorption performance. The goal of this section is to find the best design for the key parameters by performing a multi-objective optimization of the modified 3D re-entrant honeycombs, which is meaningful to decrease the value of PCS and increase the value of EA at the same time and can enhance the performance of the honeycomb under the same mass. 

### 5.1. Overall Optimization Scheme

**Figure 10:**
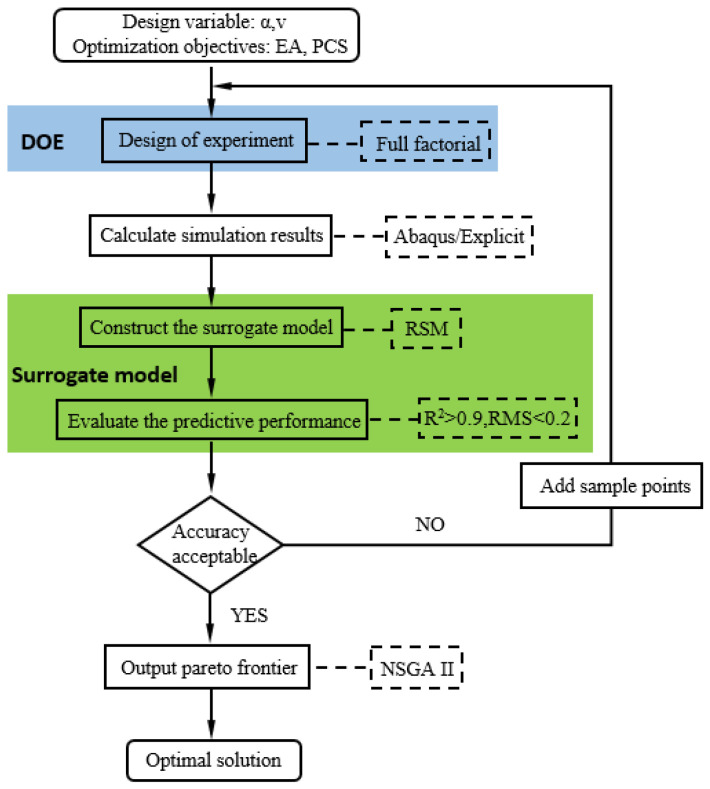
Flowchart of the multi-objective optimization process of the honeycomb.

The flowchart that illustrates the multi-objective optimization procedure for the honeycomb is presented in Figure 10. In order to accomplish this goal, the full factorial design approach (FFD) is utilized to produce appropriate sample sets after the design variables have been determined. The results are then calculated using Abaqus/Explicit for the corresponding data. Moreover, response surface methodology (RSM) is built using these results, and the surrogate model with the best accuracy is chosen using accuracy metrics like R^2^ and RMS. Lastly, optimal solutions that strike a balance between the two goals are obtained using NSGA-II in Matlab 2021. As a result, this section performs the multi-objective optimization design for the modified 3D re-entrant honeycombs to achieve the situation of optimal performance under the same mass. 

### 5.2. Design Variables and Experiments

Angle (α) and impact velocity (v) in the optimization process depicted in Figure 10 are used as design variables for the modified 3D re-entrant honeycomb. Due to the fact that there are only two variables in the design of experiment (DOE) and the interaction of them needs to be studied, the full factorial design (FFD) technique is employed in order to enhance the precision of the sample sets, resulting in the generation of 20 sample sets during the experimental design period. These sample sets serve to provide a comprehensive understanding of the design space and improve the model accuracy. Then, using the Abaqus/Explicit method, finite element models are created for each of them, and the corresponding results are calculated. Table 2 shows the sample sets for two variables as well as the results of the sample sets used in the optimization process.

### 5.3. The Surrogate Models

It is commonly known that the use of surrogate models can greatly reduce calculation time by building a mathematical model utilizing the data from sample sets. The RSM is a surrogate model that combines mathematics and mathematical statistics, and it can potentially replace complex objective functions and constraints for more straightforward ones. Using the quadratic RSM model, the approximation accuracy and computing efficiency may be well balanced. Thus, the variables and outcomes are approximated using the quadratic RSM surrogate model ultimately to construct a reasonable model in Formula (5). RSM models’ accuracy can be assessed using two distinct criteria. R-squared (R^2^) in Formula (6) is often utilized as a metric to validate the accuracy of an approximate model. Furthermore, the accuracy of the model can also be assessed using the root mean square error (RMS) in Formula (7). The following are the formulas for R^2^ and RMS:

(5) $$\bar{y}\left(x\right)=\sum_{i=1}^{N}b_{i}x_{i}+\sum_{ij}c_{ij}x_{i}x_{j}+\sum_{i=1}^{N}d_{i}{x_{i}}^{2}+e$$

(6) $$R^{2}=\frac{\sum_{i=1}^{n}{\left(y_{i}^{’}-\bar{y}\right)}^{2}}{\sum_{i=1}^{n}{\left(y_{i}-\bar{y}\right)}^{2}}=\frac{\sum_{i=1}^{n}{\left(y_{i}-\bar{y}\right)}^{2}-\sum_{i=1}^{n}{\left(y_{i}-y_{i}^{’}\right)}^{2}}{\sum_{i=1}^{n}{\left(y_{i}-\bar{y}\right)}^{2}}$$

(7) $$RMS=\sqrt{\frac{\sum_{i=1}^{n}{\left(y_{i}-y_{i}^{’}\right)}^{2}}{n-q-1}}$$

where *N* represents the number of design variables; *x_i_* and *x_j_* are set as the input variables; b, c, d, and e mean the undetermined coefficients, which can be determined using the Least Squares Method in Formula (5). In Formula (6), *y_i_* represents the ith sample point result, y’ is the computed result of ith sample point, and $\bar{y}$ denotes the mean value of the calculation results. The variables *n* and *q* represent the number of sample sets and the polynomial items in Formula (7). The surrogate model’s accuracy is generally strongly correlated with its R^2^ and RMS values. When the *RMS* is less than 0.2 and the R^2^ value is greater than 0.9, the accuracy can be deemed reasonable. 

### 5.4. Multi-Objective Optimization

In the impact process, the PCS value and the EA value are two extremely important indices. The PCS signifies the peak crushing stress within a short period of time when the upper rigid plate contacts the specimen and begins to impact, and the EA represents the energy absorption ability of the honeycomb. To further improve structural efficiency, a lower PCS value is desirable in optimization, and at the same time the EA values should be maximized when chosen as an optimization objective. It should be noted that while observing the trend of EA value and PCS value simultaneously with variable variation, the result is basically consistent, so optimizing technology is used to achieve the ideal sets to attain balanced EA and PCS values. In this section, two PCF and EA results solving the modified 3D re-entrant honeycomb using multi-objective optimization problems can be explained as follows:

(8) $$\left\{\begin{array}{l} \min\left\{PCS;1/EA\left.\right\}\right.\\ s.t.\\ 30{}^{\circ}\leq \alpha \leq 75{}^{\circ}\\ 1\;m/s\leq v\leq 70\;m/s \end{array}\right.$$

Selecting a suitable multi-objective algorithm is essential for solving the multiple target design. Therefore, the convergence and correctness of the optimized results are strongly influenced by the optimization algorithm selected. The genetic algorithm that NSGA-II is based on has the benefit of convergence [27], making it popular in engineering applications. For parameter optimization, the NSGA-II method is taken into consideration in this work.

## 6. Results and Discussion

This section mainly focuses on the optimization design in Section 5, providing optimization results and a detailed analysis and discussion of the corresponding results.

Based on the calculation and analysis results in Table 2, the final quadratic RSM surrogate model for EA and PCS can be formulated as follows:

(9) $$EA=0.0485+0.0024\alpha +0.0002\nu -1.333e^{-5}\alpha^{2}-8.802e^{-6}\nu^{2}+4.912e^{-5}\alpha \nu$$

(10) $$PCS=0.6835+0.008\alpha +0.0476\nu +1.9333e^{-4}\alpha^{2}-3.2249e^{-4}\nu^{2}+5.182e^{-5}\alpha \nu$$

**Figure 11:**
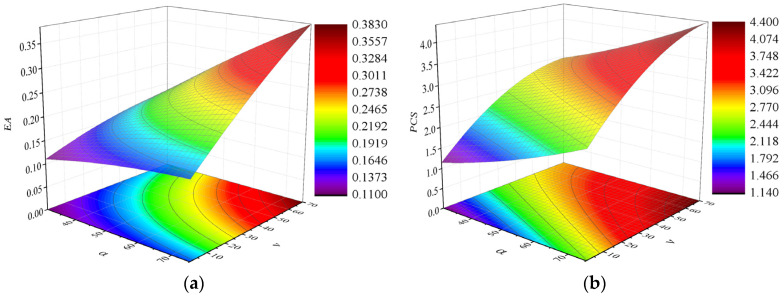
The results of the RSM: (**a**) the RSM of EA; (**b**) the RSM of PCS.

The accuracy for the final quadratic RSM model is provided in Table 3. It can be seen that the R^2^ value of EA and PCS are both greater than 0.9 and the RMS value of EA and PCS are both less than 0.2, so the accuracy of the criterion (R^2^ and RMS) can fully meet the requirements of the model. Because of its greater accuracy, the quadratic RSM model ultimately constructs a reasonable approximate model, as shown in Figure 11. It can be observed that as the value of the variables (α and v) increases, the result of optimization objectives (EA and PCS) also increases.

**Figure 12:**
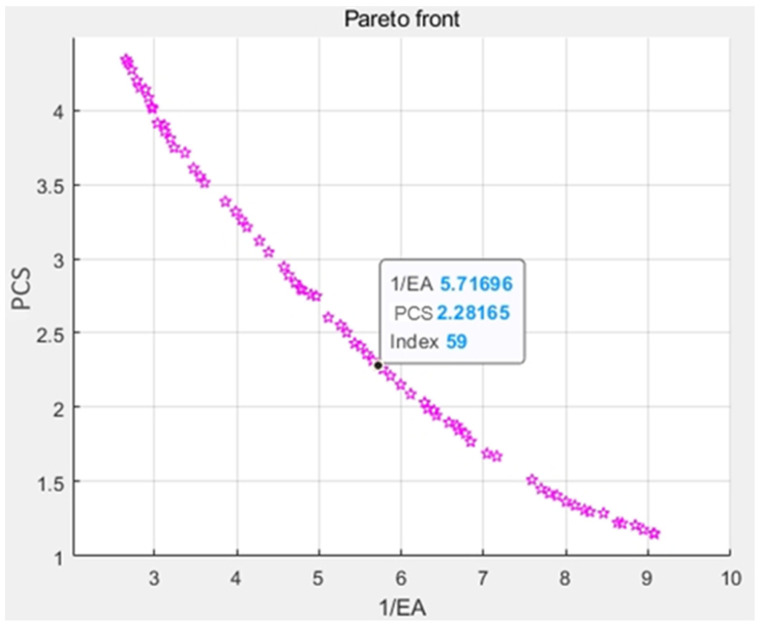
The distributions of the pareto solutions.

Figure 12 displays the yields of 70 sets of pareto optimal solutions of the NSGA-II method. It is clear from the data in Figure 11 that the PCS and EA values are in conflict with one another. These two goal parameters are unable to reach the optimum at the same time throughout the optimization process. In this study, the satisfaction function—which can be defined as follows—is used to determine the optimal solution of the NSGA-II algorithm from a set of the pareto sets by Formula (1) [26,27],

(11) $$C=\frac{PCF-PCF_{\min}}{PCF_{\max}-PCF_{\min}}+\frac{(1/EA)-{\left(1/EA\right)}_{\min}}{{\left(1/EA\right)}_{\max}-{\left(1/EA\right)}_{\min}}$$

The globally optimized value is determined by evaluating the pareto sets presented in Figure 12. When the values of 1/EA and PCS are 5.72 (1/kJ) and 2.28 MPa, and the corresponding values of α and v are 49.23° and 16.40 m/s, respectively, the minimum value is obtained by Formula (11). Subsequently, the corresponding variables of the minimum value are recalculated into the redesigned simulation model using Abaqus/Explicit. Table 4 illustrates the recalculated simulation results and the optimization outcomes achieved through the NSGA-II algorithm of the modified 3D re-entrant honeycomb, and it can be observed that the inaccuracy of EA and PCS values between the two methods are 9.26% and 0.4%, respectively; both the error ranges are within 10%. Consequently, the optimization results of NSGA-II basically agree with the results from the finite element simulation. 

It is concluded that, by utilizing the series of methods proposed in this work, the structure of the modified 3D re-entrant honeycomb can be reasonably optimized to improve the performance under the same mass conditions.

## 7. Conclusions

The impact performance of the modified 3D re-entrant honeycomb proposed in this research is thoroughly investigated using both the FE approach and an experiment. Firstly, four different angles of modified 3D re-entrant honeycomb with the same mass were designed and fabricated by the LCD method. The deformation modes and dynamic responses of the honeycomb are investigated by sample experiments, and an FE model is established using parametric modeling to study the stress–strain characteristic and energy absorption of the honeycomb at different angles and velocities. In addition, an optimization to balance EA and PCS is carried out.

(1) Through the experiment, it can be revealed that four different angles of modified 3D re-entrant honeycomb all exhibit NPR phenomena. With the increase in the honeycomb angle, the porous voids of the honeycomb increase. Under the same external compression, the force is less likely to be transmitted to the lower part of the honeycomb. Additionally, the truss inside the honeycomb structure becomes increasingly unstable even to collapse during the compression process, emphasizing the negative Poisson’s ratio phenomena.
(2) The FE results show that when the honeycomb angle is 45°, the maximum stress is relatively lower under different velocity conditions. Overall, as the honeycomb angle increases, the impact stress steadily decreases, and the EA value increases simultaneously. When the honeycomb angle remains constant, both the impact stress and EA value increase with the increase in impact velocity.
(3) The pareto solution set is obtained through the NSGA-II algorithm, and the minimum value of C is selected as the best solution from the solution set. C is obtained when the values of 1/EA and PCS are 5.72 (1/kJ) and 2.28 MPa, and the corresponding values of α and v are 49.23° and 16.40 m/s, respectively. According to the FE verification calculation, the relative errors of EA and PCS values between the optimization values are 9.26% and 0.4%, respectively. Consequently, the optimization approach is efficient and a useful model for optimizing the design of corresponding honeycomb structures under the same mass.

## Figures and Tables

**Table 1:** The parameters of four modified 3D re-entrant honeycombs.

α	30°	45°	60°	75°
L_x_ (mm)	66.144	68.688	72.0	75.86
L_z_ (mm)	66.144	68.688	72.0	75.86
L_y_ (mm)	32.0	35.312	37.856	39.456
L_1_ (mm)	1.5	1.5	1.5	1.5
L_2_ (mm)	2	2	2	2
L_3_ (mm)	1.5	1.5	1.5	1.5
d (mm)	2	2	2	2
t (mm)	0.8	0.8	0.8	0.8

**Table 2:** Sample sets and results of variables.

	PCS (MPa)	EA (kJ)	PCS (MPa)	EA (kJ)	PCS (MPa)	EA (kJ)	PCS (MPa)	EA (kJ)
v/α	30°		45°		60°		75°	
1 m/s	1.20	0.12	1.42	0.13	1.99	0.14	2.10	0.14
10 m/s	1.82	0.13	1.72	0.16	2.24	0.18	3.22	0.20
30 m/s	2.20	0.14	2.68	0.17	3.10	0.25	3.79	0.28
50 m/s	2.40	0.17	3.19	0.21	3.6	0.28	4.10	0.33
70 m/s	3.10	0.19	3.45	0.26	3.9	0.34	4.23	0.36

**Table 3:** The accuracy of the RSM model.

	EA	PCS
R^2^ (>0.9)	0.974	0.919
RMSE (<0.2)	0.060	0.105

**Table 4:** The accuracy of NSGA-II.

	NSGA II	Simulation	Error
1/EA	5.72	6.25	9.26%
PCS	2.28	2.27	0.4%

## Data Availability

The original contributions presented in this study are included in this article, further inquiries can be directed to the corresponding author.
